# Thalamic nuclei volumes are related to disease stage in patients with amyotrophic lateral sclerosis

**DOI:** 10.3389/fnins.2025.1616239

**Published:** 2025-07-11

**Authors:** Tianrui Wen, Jun Zhu, Sujuan Sun, Yujing Chen, Ninglu Gao, Mingjie Ma, Xinyue Chen, Shuangwu Liu, Pengfei Lin, Yan Deng

**Affiliations:** ^1^School of Medicine, Cheeloo College of Medicine, Shandong University, Jinan, China; ^2^Shandong Provincial Key Laboratory of Mitochondrial Medicine and Rare Diseases, Department of Neurology, Research Institute of Neuromuscular and Neurodegenerative Diseases, Qilu Hospital of Shandong University, Jinan, China; ^3^School of Nursing and Rehabilitation, Cheeloo College of Medicine, Shandong University, Jinan, China; ^4^Department of Radiology, Qilu Hospital of Shandong University, Jinan, China

**Keywords:** amyotrophic lateral sclerosis, thalamus, MRI, bilateral anteroventral, bilateral pulvinar-limitans

## Abstract

**Objective:**

To explore atrophy patterns in thalamic nuclei at different phases of amyotrophic lateral sclerosis (ALS) and determine any correlations between thalamic nucleus volume and either cognitive impairments or motor disabilities.

**Methods:**

We used the King’s clinical staging system for ALS to divide 76 consecutive patients with ALS by disease stage. We investigated patterns of thalamic atrophy in the patients and in 94 healthy controls (HCs). Cognitive functions were evaluated with the Mini-Mental State Examination (MMSE), Frontal Assessment Battery, Boston Naming Test, and Auditory Verbal Learning Test.

**Results:**

Considering all ALS patients, no significant differences were observed in the volume of any thalamic nuclei between the ALS group and HCs. Thalamic nucleus volumes remained normal in ALS patients at King’s Stage 2 and Stage 3. However, atrophy was detected in the bilateral anteroventral nucleus, bilateral pulvinar-limitans, bilateral mediodorsal-paratenial-reuniens, bilateral motor hub, bilateral sensory hub, and bilateral intralaminar nucleus in patients who had reached King’s Stage 3. In these patients, the volume of the bilateral motor nuclei was associated with the revised ALS Functional Rating Scale scores, and that of the right pulvinar-limitans independently correlated with MMSE scores.

**Conclusion:**

Our study provides a comprehensive profile of thalamic atrophy in ALS patients. The thalamic atrophy patterns in these patients extremely differs at different King’s Stages, and we suggest that these alterations might result largely from sequential, regional patterns of TDP-43 pathology in ALS. Furthermore, thalamic atrophy might play important roles in motor disability and global cognitive impairments observed in patients with ALS.

## Introduction

Amyotrophic lateral sclerosis (ALS) is a rare neurodegenerative disease with both clinical and hereditary heterogeneity (Swinnen and Robberecht, 2014; Taylor et al., 2016; Eisen et al., 2017). ALS is likely derived from cortical influences, selectively damaging the anterior horn cells of the spinal cord, motor nuclei of the brainstem and pyramidal tracts, etc., resulting in asymmetric muscle weakness and muscle atrophy starting from the distal limbs in patients and is currently considered a multisystemic disorder in which almost half of patients present with varying degrees of cognitive deficits (Eisen et al., 2017; Braak et al., 2013). The high differentiation and complexity of the human CM system make it more susceptible to the pathology of 43 kDa transactive response DNA-binding protein (TDP-43) pathology in ALS is typically detected in the motor cortex at early Braak stages, with sequential spread to the prefrontal cortex, thalamus, and hippocampus. The resulting corticospinal tract damage underlies asymmetric muscle weakness and distal limb atrophy (Eisen et al., 2017; Braak et al., 2013; Brettschneider et al., 2013; Kassubek et al., 2014).

The thalamus is a crucial relay center of the brain, comprising multiple highly specific nuclei (Sherman, 2016). Neuronal populations located in distinct nuclei of the thalamus are associated with various functional specializations, and are connected to different cortical areas (Sherman, 2016; Jaramillo et al., 2019). Notably, neuroimaging studies of thalamic volume in patients with ALS have so far been largely contradictory, with reports of both no differences and smaller volumes, compared with healthy individuals (Finegan et al., 2019; Tae et al., 2020; Westeneng et al., 2015). Recently, Chipika et al. suggested that the controversy is rooted in the averaging of biophysical indices across affected and unaffected thalamic nuclei (Chipika et al., 2020). However, according to Braak stages, TDP-43 pathology in ALS is not typically detected in the large neurons of thalamic nuclei until Braak stage 2 (Brettschneider et al., 2013). Thus, thalamic alterations are not likely to be an early feature of ALS.

The current study had two aims. First, using a relatively large sample, we wished to verify whether selective atrophy of thalamic nuclei occurs in Chinese patients with ALS. We further hypothesized that volumetric alterations in these susceptible thalamic nuclei would emerge only at advanced disease phases. Thus, we used the well-validated King’s clinical staging system for ALS to explore atrophy patterns in thalamic nuclei at different disease stages using *in vivo* structural MRI (Roche et al., 2012). Second, the thalamus is known to be essential for cognition (Sherman, 2016; Behrens et al., 2003). Although few studies have focused on this topic in ALS, thalamic abnormalities are likely involved in the cognitive impairment observed in older individuals and in other neurodegenerative diseases (Low et al., 2019; Bocchetta et al., 2020; Aggleton et al., 2016). Thus, in the present study, we sought to explore distinct thalamic atrophy-related correlates of cognitive impairment and motor disability in patients with ALS.

## Materials and methods

### Participants

Seventy-eight newly diagnosed patients with ALS were consecutively included in the study between November 2019 and November 2020. All patients met the revised El Escorial criteria for possible, probable, or definite ALS. All patients were verified as presenting with progressive disability during a three-month telephone follow-up visit. The exclusion criteria for the patients were as follows: (1) family history of ALS; (2) inability to complete an MRI scan; (3) comorbid frontotemporal dementia (FTD); (4) comorbid with other neurological or psychiatric conditions, and (5) refused to participate. We used the Rascovsky criteria to diagnose FTD (Rascovsky et al., 2011). Additionally, 94 age-matched healthy controls (HCs) were also recruited from community. HCs were subjected to the same exclusion criteria as ALS patients.

### Clinical screening

We recorded all demographic and clinical information, including age, sex, education, family history of neurological disease, comorbid conditions, site of symptom onset, and disease duration. The revised ALS Functional Rating Scale (ALSFRS-R) was used to assess disease severity (Cedarbaum et al., 1999). Depression and anxiety were quantified using the Hamilton Depression Rating Scale (HDRS) and the Hamilton Anxiety Rating Scale (HARS), respectively (Liu et al., 2018). The information was then corroborated by an informant (the patient’s spouse, relative, or primary caregiver).

### Neuropsychological evaluation

The patients completed a neuropsychological test battery to screen for cognitive and behavioral features (Liu et al., 2018; Pan et al., 2020). Briefly, the screening battery included the Mini-Mental State Examination (MMSE), Frontal Assessment Battery (FAB), Boston Naming Test (BNT), and Auditory Verbal Learning Test (AVLT). Behavioral symptoms were assessed through an interview with the informant and quantified using the Frontal Behavioral Inventory (FBI).

### ALS staging

During clinical screening, clinical staging was evaluated using King’s clinical staging system (Roche et al., 2012). Stages 1–3 of the disease are based on the body regions involved (bulbar, upper limbs, and lower limbs), and Stage 4 is defined as the necessity of nutritional or respiratory support. The King’s staging system might be closely linked to anatomical spread. Because the naming of the Stage 4 milestones is potentially problematic, patients with ALS have demonstrated less homogeneity between Stage 4 and the other three stages, and only two patients in this cohort were classified as King’s Stage 4, we opted not to include Stage 4 in the final analysis.

### MRI acquisition

All MRI data were obtained on a 3.0 T magnetic resonance system (Philips Medical System Ingenia scanner) with dStream head coil. During the scan, all subjects were asked to be quiet, remain supine, and refrain from any conscious thinking. Structural images of the whole brain were scanned using a three-dimensional (3D) fast spoiled gradient-echo sequence: repetition time (TR) = 6.7 ms, echo time (TE) = 3.0 ms, matrix = 68 × 68, voxel size = 1 mm × 1 mm × 1 mm, field of view (FOV) = 240 mm × 240 mm, slice thickness = 1.0 mm, no slice gap, and a total of 180 slices. FLAIR data were scanned using TR = 7,000 ms, Flip Angle 90°, TE = 125 ms, acquisition matrix = 272 × 176, and slice thickness 6 mm.

### Thalamic nuclei segmentation

**Figure 1:**
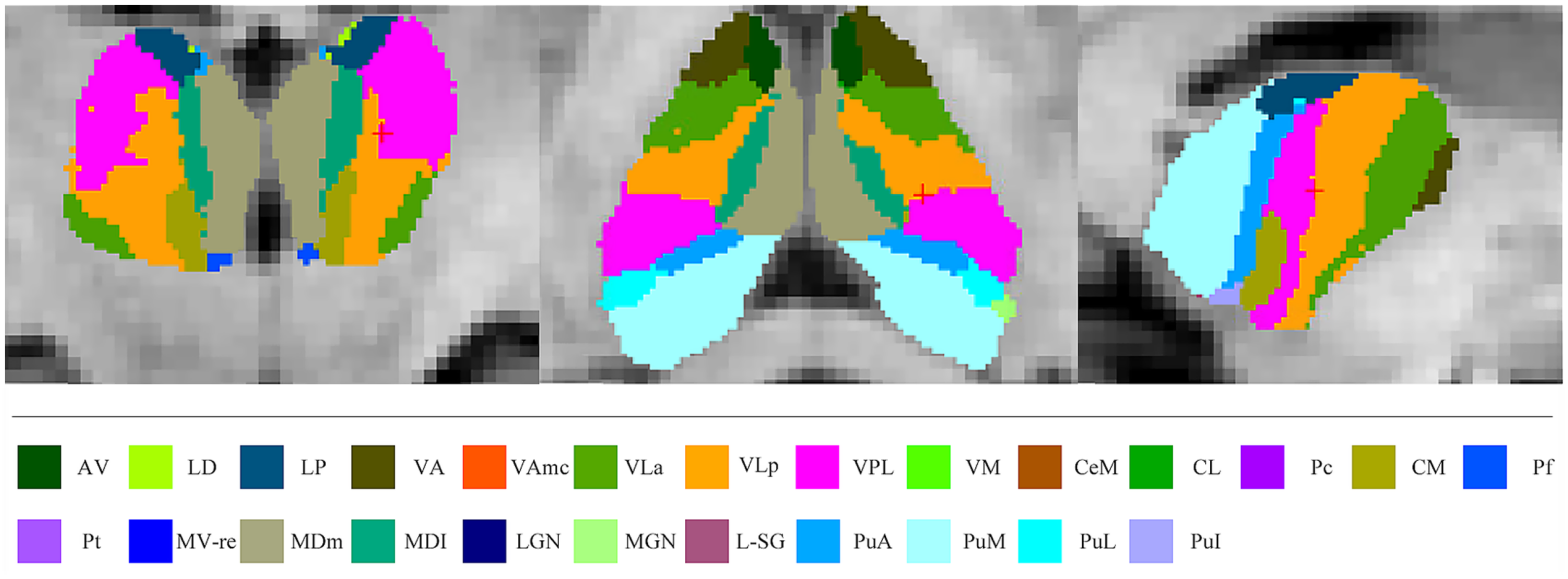
Atlas-based segmentation of the thalamus. AV, anteroventral; LD, laterodorsal; LP, lateral posterior; VA, ventral anterior; VAmc, ventral anterior magnocellular; VLa, ventral lateral anterior; VLp, ventral lateral posterior; VPL, ventral posterolateral; VM, ventromedial; CeM, central medial; CL, central lateral; Pc, paracentral; CM, centromedian; Pf, parafascicular; Pt, paratenial; MV-re, reuniens medial ventral; MDm, mediodorsal medial magnocellular; MDl, mediodorsal lateral parvocellular; LGN, lateral geniculate; MGN, medial geniculate; L-SG, limitans-suprageniculate; PuA, pulvinar anterior; PuM, pulvinar medial; PuL, pulvinar lateral; PuI, pulvinar inferior.

In the present study, thalamic nuclei were automatically segmented and measured using FreeSurfer version 7.1.0.^1^ A package available in FreeSurfer 7.1.0 was able to automatically segment the thalamic nuclei (Iglesias et al., 2018). Using this algorithm, the thalamus was accurately segmented into the following nuclei: anteroventral (AV), laterodorsal (LD), lateral posterior (LP), ventral anterior (VA), ventral anterior magnocellular (VAmc), ventral lateral anterior (VLa), ventral lateral posterior (VLp), ventral posterolateral (VPL), ventromedial (VM), central medial (CeM), central lateral (CL), paracentral (Pc), centromedian (CM), parafascicular (Pf), paratenial (Pt), reuniens (medial ventral) (MV-re), mediodorsal medial magnocellular (MDm), mediodorsal lateral parvocellular (MDl), lateral geniculate (LGN), medial geniculate (MGN), limitans-suprageniculate (L-SG), pulvinar anterior (PuA), pulvinar medial (PuM), pulvinar lateral (PuL), and pulvinar inferior (PuI) (Figure 1) (Iglesias et al., 2018). The procedure, which included motion correction, intensity normalization, automated topology corrections, and the automatic segmentation of cortical and subcortical regions, has been documented in detail elsewhere (Chipika et al., 2020). Total intracranial volume (TIV) was calculated for each participant for further analysis as a covariate. FreeSurfer segmentation follows the standard quality control of the software and verifies the results through repeated measurements to ensure reliability.

Several nuclei, including some relevant to ALS, were not examined because of their small size or lack of contrast with surrounding white matter. These included the laterodorsal, lateral, and medial geniculate nuclei. The remaining thalamic nuclei were merged into seven core groups based on their distinct physiological function (Table 1) (Chipika et al., 2020; Iglesias et al., 2018). In the current study, we limited analysis to these core groups because of their reliable segmentation and putative involvement in ALS.

**Table 1:** Thalamic core group.

Thalamic core group	Thalamic nuclei
Anteroventral	AV
Pulvinar-limitans	PuA, PuM, PuL, PuI, L-SG
Lateroposterior	LP
Mediodorsal-paratenial-reuniens	MDm, MDl, MV-re, Pt
Motor hub	VA, VAmc, VLa, VLp
Sensory hub	VPL, VM
Intralaminar	CeM, CL, Pc, CM, Pf

AV, anteroventral; LD, laterodorsal; LP, lateral posterior; VA, ventral anterior; VAmc, ventral anterior magnocellular; VLa, ventral lateral anterior; VLp, ventral lateral posterior; VPL, ventral posterolateral; VM, ventromedial; CeM, central medial; CL, central lateral; Pc, paracentral; CM, centromedian; Pf, parafascicular; Pt, paratenial; MV-re, reuniens medial ventral; MDm, mediodorsal medial magnocellular; MDl, mediodorsal lateral parvocellular; LGN, lateral geniculate; MGN, medial geniculate; L-SG, limitans-suprageniculate; PuA, pulvinar anterior; PuM, pulvinar medial; PuL, pulvinar lateral; PuI, pulvinar inferior.

### Ethical approval

This study was approved by the Research Ethics Committee of the School of Medicine, Shandong University. Participant information was only collected after all patients and HCs were made aware of the purpose of the study and provide informed written consent.

### Statistical analysis

#### Clinical data analysis

Continuous variables are reported as the mean and standard deviation, whereas categorical variables are reported as the frequency and proportion. Student’s *t*-tests or analysis of variance (ANOVA) were used to compare continuous variables (with Mann–Whitney *U* tests as necessary). Categorical variables were compared using chi-squared tests. Bonferroni-corrected *post hoc t*-tests were performed to identify pairwise group differences. Bonferroni correction is a conservative method that adjusts the significance threshold by dividing the alpha level (*α* = 0.05) by the number of tests, which is ideal for confirmatory research where false positives must be minimized. *p*-values < 0.05 indicated significance. All statistical analyses were performed using SPSS version 20.0 (IBM Corp., Armonk, NY).

#### MRI data analysis

For each GM structure, analysis of variance (ANOVA) models was constructed to investigate differences in the structural volumes maps between groups. We used age, sex, and TIV as covariates. To identify pairwise group differences, further *post hoc t*-tests were performed between groups. After Bonferroni correction, *p* < 0.05 was taken as significant. Partial correlations were performed between the imaging metrics and the clinical data, controlling for age, sex, and TIV. To avoid type II errors, the partial correlations were restricted to clinical data and imaging metrics that were significantly different between groups. Values of *p* < 0.05 were recognised as significant.

## Results

### Demographic and clinical information

Finally, seventy-six consecutive patients with ALS and 94 HCs were included in the present study. All ALS patients completed the clinical screening, MRI acquisition, behavior, anxiety and depression assessment. A total of 76 ALS patients completed the cognitive test battery while the remaining two patients were unable to due to severe physical disability. MMSE and FAB scores were lower in the patients than in HCs (*p* < 0.05). HDRS and HARS scores were higher in the patients than in HCs (*p* < 0.05). Demographic and clinical information for patients and HCs are shown in Table 2.

**Table 2:** Demographic and clinical features of patients with ALS and HCs.

Variable	Patients with ALS (*n* = 76)	HCs (*n* = 94)	*p*-value
Age (years)	57.5 ± 11.3	55.2 ± 6.8	0.11
Men/Women (*n*)	43/33	35/59	0.01
Education	9.5 ± 4.0	10.3 ± 3.8	0.17
ALS duration (month)	11.6 ± 7.5	–	–
Bulbar ALS onset *n*, (%)	17 (22.3)	–	–
ALSFRS-R score	41.4 ± 3.2	–	–
Riluzole, *n* (%)	5 (6.5)	–	–
King’s clinical stage (stages 1/2/3), %	26.3, 52.6, 21.0%	–	–
MMSE	26.6 ± 3.0	28.3 ± 1.9	<0.01
FAB	14.6 ± 2.0	17.2 ± 0.7	<0.01
FBI	1.2 ± 1.4	–	–
BNT	23.7 ± 4.2	24.9 ± 4.1	0.16
AVLT, short delayed (5 min)	7.2 ± 3.1	7.7 ± 3.3	0.24
AVLT, long delayed (20 min)	6.5 ± 3.3	7.4 ± 2.8	0.06
HARS	8.1 ± 4.6	2.6 ± 3.7	<0.01
HDRS	11.4 ± 7.1	3.4 ± 3.8	<0.01

ALS, amyotrophic lateral sclerosis; HC, healthy control; ALFRS-R, ALS Functional Rating Scale-Revised; MMSE, Mini-Mental State Examination; FAB, Frontal Assessment Battery; FBI, Frontal Behavioral Inventory; BNT, Boston Naming Test; AVLT, Auditory Verbal Learning Test; HARS, Hamilton Anxiety Rating Scale; HDRS, Hamilton Depression Rating Scale.

### Findings among patients with ALS at different King’s clinical stages

On the basis of the body regions involved, patients with ALS were divided into corresponding King’s clinical stages at clinical screening. No significant differences were found among the three patient subgroups for most information, except for age and ALSFRS-R score. Post-hoc analysis shown that patients at King’s Stage 3 were significantly older than those at King’s Stage 1. Moreover, ALSFRS-R scores also differed significantly according to King’s stage. Demographic and clinical information for each disease-stage group are shown in Table 3.

**Table 3:** Demographic and clinical information for each disease stage subgroup.

Variable	Stage 1 (*n* = 20)	Stage 2 (*n* = 40)	Stage 3 (*n* = 16)	*F* or χ^2^	*p*-value
Age (years)	53.7 ± 12.3	57.0 ± 10.8	63.2 ± 9.5	3.45	0.04
Men/Women (*n*)	11/9	25/15	7/9	1.66	0.43
Education	10.3 ± 3.9	9.1 ± 4.1	9.5 ± 3.6	0.53	0.59
ALS duration (month)	9.1 ± 4.1	12.1 ± 8.5	13.1 ± 7.6	1.55	0.22
Bulbar ALS onset *n*, (%)	5 (25)	9 (22.5)	3 (18.7)	0.07	0.96
ALSFRS-R score	44.5 ± 1.4	41.3 ± 2.3	37.8 ± 2.9	38.05	<0.01
MMSE	27.4 ± 2.6	26.4 ± 2.6	26.1 ± 4.1	1.02	0.36
FAB	15.1 ± 1.1	14.7 ± 2.1	13.8 ± 2.4	1.85	0.17
BNT	25.2 ± 4.8	23.3 ± 3.8	22.8 ± 4.1	1.84	0.16
AVLT, short delayed	7.9 ± 3.2	7.0 ± 2.9	6.8 ± 3.2	0.68	0.51
AVLT, long delayed	7.2 ± 3.5	6.2 ± 3.3	6.1 ± 3.2	0.67	0.50
HARS	8.7 ± 4.8	7.6 ± 4.6	8.7 ± 4.0	0.44	0.64
HDRS	12.4 ± 9.8	10.5 ± 5.9	12.3 ± 5.4	0.63	0.53

ALS, amyotrophic lateral sclerosis; HC, healthy control; ALFRS-R, ALS Functional Rating Scale-Revised; MMSE, Mini-Mental State Examination; FAB, Frontal Assessment Battery; FBI, Frontal Behavioral Inventory; BNT, Boston Naming Test; AVLT, Auditory Verbal Learning Test; HARS, Hamilton Anxiety Rating Scale; HDRS, Hamilton Depression Rating Scale.

### Volumes of thalamic nuclei

No significant differences were observed in thalamic nuclei volumes between the whole ALS group and HCs. However, significant differences were found for all nuclei except the right lateroposterior nucleus when comparing across ALS King’s stage subgroups and HCs (Table 4).

**Table 4:** Profiles of the thalamic nuclei at each ALS disease stage and for HCs.

Thalamic nuclei	HCs (*n* = 94)	Stage 1 (*n* = 20)	Stage 2 (*n* = 40)	Stage 3 (*n* = 16)	*F*	*p*-value
Left thalamus, mm^3^						
Anteroventral	113.1 ± 18.9	113.6 ± 16.9	110.6 ± 20.0	99.0 ± 12.4	2.75	0.04
Pulvinar-limitans	1772.5 ± 189.2	1829.3 ± 216.2	1783.2 ± 240.8	1597.7 ± 176.7	4.36	<0.01
Lateroposterior	102.6 ± 17.9	109.1 ± 20.3	102.0 ± 17.5	91.3 ± 20.2	2.83	0.04
Mediodorsal-paratenial-reuniens	989.2 ± 104.6	990.8 ± 94.6	984.9 ± 125.8	893.6 ± 80.7	3.79	0.01
Motor hub	1708.1 ± 199.2	1716.7 ± 184.6	1709.0 ± 186.3	1473.2 ± 156.8	7.40	<0.01
Sensory hub	852.8 ± 102.3	861.8 ± 107.2	836.4 ± 99.1	732.8 ± 88.9	6.82	<0.01
Intralaminar	373.8 ± 42.9	378.9 ± 48.5	367.1 ± 46.2	325.0 ± 35.3	6.10	<0.01
Global thalamus	6273.8 ± 671.2	6368.9 ± 678.5	6248.7 ± 696.4	5525.4 ± 523.1	6.90	<0.01
Right thalamus, mm^3^						
Anteroventral	118.9 ± 16.6	121.6 ± 14.6	118.3 ± 17.9	106.0 ± 23.7	2.82	0.03
Pulvinar-limitans	1646.0 ± 193.2	1654.7 ± 249.4	1650.6 ± 227.2	1474.7 ± 193.2	3.47	0.01
Lateroposterior	101.4 ± 17.1	106.6 ± 19.4	105.2 ± 15.6	94.1 ± 12.9	2.23	0.08
Mediodorsal-paratenial-reuniens	983.8 ± 99.8	968.4 ± 110.2	973.2 ± 140.1	868.5 ± 119.7	4.73	<0.01
Motor hub		1718.7 ± 198.9	1702.2 ± 215.2	1450.6 ± 168.6	7.56	<0.01
Sensory hub		827.9 ± 108.5	822.7 ± 109.4	699.3 ± 75.8	6.89	<0.01
Intralaminar		373.5 ± 47.5	369.8 ± 48.0	324.7 ± 36.1	5.37	<0.01
Global thalamus		6131.1 ± 708.5	6105.4 ± 730.6	5331.4 ± 530.6	6.67	<0.01

**Figure 2:**
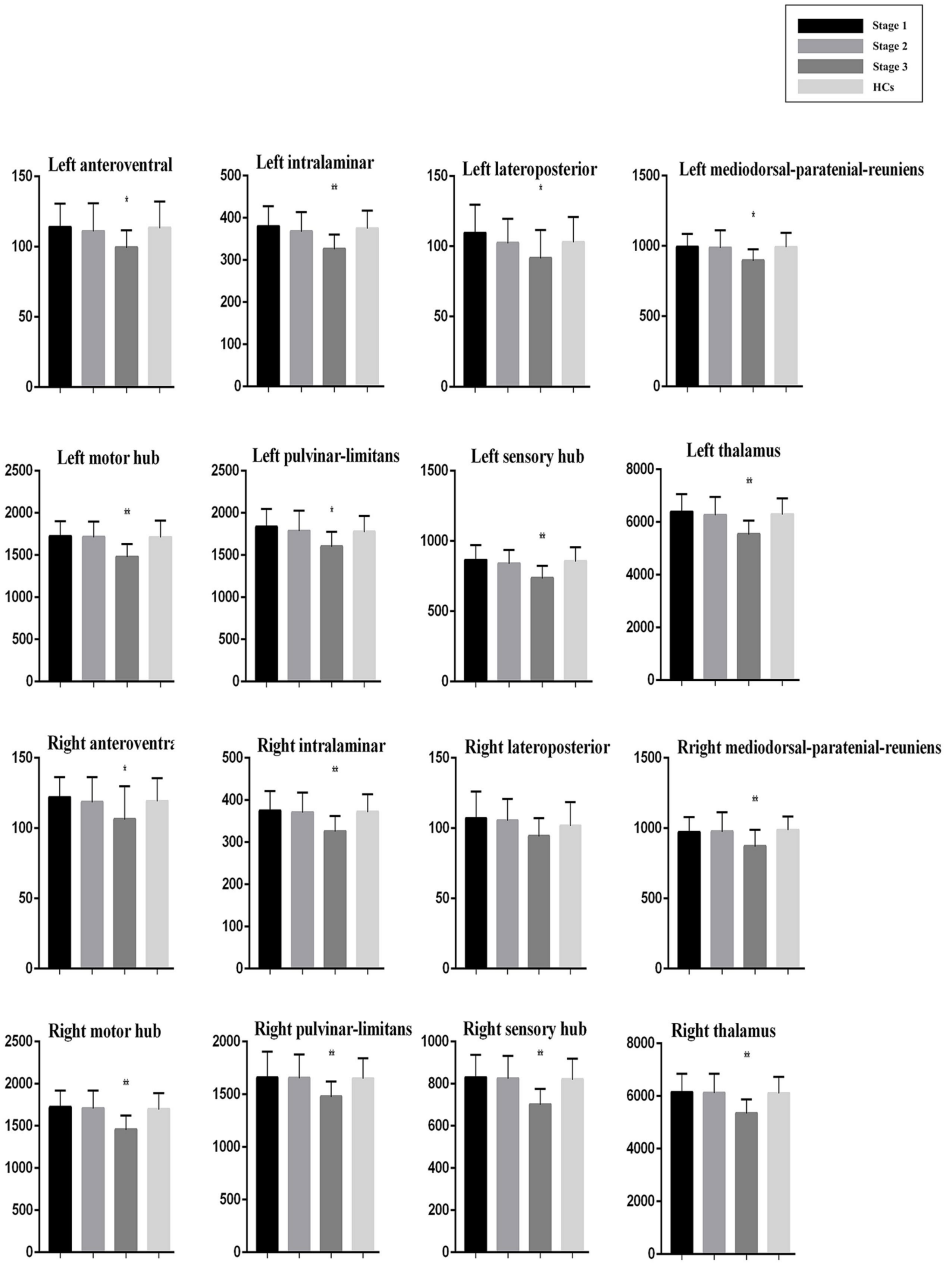
Thalamic nuclei profiles for patients with ALS at each disease stage and HCs. Volume (mm^3^).

*Post hoc* analysis showed that compared with HCs, ALS patients at King’s Stage 3 had reduced volumes for the bilateral anteroventral, bilateral pulvinar-limitans, bilateral mediodorsal-paratenial-reuniens, bilateral motor hub, bilateral sensory hub, bilateral intralaminar, and bilateral global thalamus groups after Bonferroni correction. No significant differences in thalamus volumes were observed between the HCs and patients with ALS at King’s Stage 1 or Stage 2. Compared with patients at King’s Stage 1, those at King’s Stage 3 had less volume in the left pulvinar-limitans, left mediodorsal-paratenial-reuniens, bilateral motor hub, bilateral sensory hub, bilateral intralaminar, and bilateral global thalamus groups after Bonferroni correction. Compared with patients at King’s Stage 2, those at King’s Stage 3 had less volume in the bilateral pulvinar-limitans, bilateral mediodorsal-paratenial-reuniens, bilateral motor hub, bilateral sensory hub, bilateral intralaminar, and bilateral global thalamus groups after Bonferroni correction. Profiles of the thalamic nuclei for HCs and patients with ALS at each disease stage are presented in Figure 2.

### Correlation analyses

There were no significant correlations between thalamic nucleus volume and clinical data for patient at King’s Stage 1 or 2. However, for patients at King’s Stage 3, we found a significant correlation between ALSFRS-R and left (*r* = 0.517, *p* = 0.04), and right (*r* = 0.544, *p* = 0.03) motor nuclei volumes. Moreover, right pulvinar-limitans volume significantly correlated with MMSE score (*r* = 0.584, *p* = 0.02). We did not find any significant correlations between thalamic nuclei volumes and disease course, FAB score, BNT score, HARS score, or HDRS score.

## Discussion

In a relatively large cohort of patients with ALS, with a state-of-the-art thalamic segmentation approach, we demonstrated that the pattern of thalamic atrophy observed in patients with ALS extremely differed at different King’s clinical disease stages. We found that thalamic atrophy, either global or at specific nuclei, did not emerge until King’s Stage 3. Furthermore, we also found that after controlling for age, sex, and TIV, for patients at Stage 3, reduced volume in bilateral thalamic motor nuclei was associated with greater disease severity, and reduced volume in the right pulvinar-limitans was independently correlated with global cognitive deficit. Thus, thalamic atrophy may play pivotal roles in the motor disabilities and cognitive impairments in ALS. Our findings align with the perspective that ALS is a multisystemic neurodegenerative disorder, with prior neuropathology studies suggesting that TDP-43 pathology may originate in the motor cortex and spread in a sequential, regional pattern. The thalamic atrophy patterns observed in Stage 3 patients further support this model of neurodegenerative spread, even as our study focuses on subcortical changes (Brettschneider et al., 2013).

Although thalamus abnormalities in patients with ALS have been extensively detected by dedicated metabolic and functional imaging studies, volumetric atrophy of the thalamus detected by structural MRI in patients with ALS remain largely contradictory. Some studies have reported no differences, while others have reported smaller volumes, and still others have reported focal atrophy limited to specific thalamic nuclei (Finegan et al., 2019; Tae et al., 2020; Westeneng et al., 2015; Sharma et al., 2011; Chiò et al., 2014). Brettschneider and colleagues reported that TDP-43 pathology, which are the signal of neuronal damage in patients with ALS, can be divided into four stages according to their range and severity (Braak stages) (Brettschneider et al., 2013). Braak stage 2 is characterized by TDP-43 pathology in the prefrontal areas, reticular formation, and precerebellar nuclei (Brettschneider et al., 2013). Moreover, the large neurons of the thalamic nuclei also develop TDP-43 pathology at this stage (Brettschneider et al., 2013). Thus, according to the Braak stage, TDP-43 pathology-related thalamic alterations emerge at Stage 2 (Brettschneider et al., 2013). In line with neuropathology studies, our findings further demonstrated that atrophy of thalamic nuclei in patients with ALS did not emerge until King’s Stage 3. Overall, our data and those of others suggest that thalamic alterations are not likely to be an early feature of ALS. The controversy regarding thalamic atrophy in previous neuroimaging studies of ALS was likely caused by averaging biophysical indices of patients across affected and unaffected disease stages (Kassubek et al., 2014; Finegan et al., 2019; Tae et al., 2020; Westeneng et al., 2015). For example, global thalamic volume without nuclear segmentation, which masks regional atrophy (Westeneng et al., 2015), pooled measurements of functionally distinct nuclei (e.g., merging motor and sensory hubs), ignoring regional vulnerability to TDP-43 pathology (Chipika et al., 2020).

Indeed, our findings demonstrated that neither global thalamus volume nor the volumes of specific thalamic nuclei, differed significantly between the ALS group as a whole and HCs. However, when we used the well-validated King’s clinical staging system to divide ALS patients into disease-stage subgroups, we found that although thalamic volume remained normal for patients at King’s Stage 1 and 2, global thalamic volume was reduced in patients at King’s Stage 3, as were the volumes of a great number of individual nuclei. Recently, Tae and colleagues also reported that thalamic volumes did not differ significantly between patients with ALS and HCs in a group of patients at relatively early-phase ALS (disease duration of only 12.99 months) (Tae et al., 2020). Westeneng and colleagues reported that even though patients with ALS did not exhibit reduced thalamic volume at baseline compared with HCs, thalamic volume tended to be reduced after a 5-month follow-up (Westeneng et al., 2015). However, they did not analyze the volumes of individual thalamic nuclei. Chipika and colleagues reported the involvement of ventral lateral, ventral anterior, mediodorsal-paratenial-reuniens, and sensory thalamic nuclei in a group of patients with advanced-stage ALS (mean ALSFRS-R scores of 36.6), suggesting the preferential involvement of specific nuclei rather than global thalamic atrophy in patients with ALS (Chipika et al., 2020). These studies further support our viewpoint, although they do not divide patients into subgroups according to disease stages.

The thalamus is well known to participate in many different neuronal pathways, with functions that are not restricted to motor behavior, including those related to emotional and cognitive abilities (Sherman, 2016; Behrens et al., 2003). Atrophy in distinct thalamic nuclei might contribute to phenotype-defining cognitive, mood, and motor deficits, however, few studies have focused on these relationships in patients with ALS (Sherman, 2016; Jaramillo et al., 2019). In a group of 20 patients with ALS, Tu and colleagues used diffusion MRI to show that widespread changes in diffusion patterns in motor and extramotor thalamic regions, as well as diffusivity measures, were significantly correlated with disease duration and ALSFRS-R score (Tu et al., 2018). Recently, Chipika and colleagues found a significant association between pulvinar volume and disease duration, but no direct correlations between motor nuclei volume and ALSFRS-R score (Chipika et al., 2020). The current study further demonstrates that the volume of thalamic motor nuclei is associated with ALSFRS-R scores in patients with ALS. Foremove, previous studies did not establish the relations between specific thalamic nuclei volumes and cognitive impairments in ALS patients (Finegan et al., 2019; Tae et al., 2020; Westeneng et al., 2015). In particular the thalamus is extensively connected to cerebral cortex. and play a key role in cognition (Sherman, 2016).

Thus, one of the key findings of this study was to demonstrate volume reduction of the specific thalamic nuclei are significantly associated with cognitive deficits in patients with ALS. In the present study, the volume of the right pulvinar-limitans nucleus was associated with global cognitive functions in the patients with ALS. In line with our study, in a large cohort of patients with psychotic disorders, Huang and colleagues also found that global cognitive function was associated with pulvinar volume rather than mediodorsal nuclei volumes (Huang et al., 2020). Indeed, the pulvinar is a crucial region of the limbic system, and might play a physiological role in attentional processing, working memory, and decision making. Effective connections between cortical areas could be gated by the pulvinar, and, thus, pulvinar lesions could influence cognitive functions through the pulvino-cortical circuits (Jaramillo et al., 2019). Moreover, recent studies show that cognitive impairment might worsen across King’s stages in patients with ALS (Chiò et al., 2019). Our findings provide evidence that supports these studies and further highlights the fact that cognitive deficits do not rely solely on cortical integrity, but also depend on the thalamus in ALS. Thus, the thalamus is critical and its degeneration in patients with ALS may underlie some of the observed cognitive impairments (Gregory et al., 2020; Machts et al., 2015).

The present study had several limitations. First, we used a cross-sectional design, preventing the establishment of causality between thalamic atrophy, motor disability, and cognitive deficits. Second, our results were susceptible to selection bias because the patients who visit our center commonly have a relatively short disease course (we are the largest ALS center in the Shandong province). Third, our study only used structural MRI to explore the changes of thalamic gray matter. Further studies are still needed to verify whether our findings on thalamic atrophy patterns and stage-dependent correlations are similar to those obtained via other imaging modalities (e.g., functional MRI, diffusion tensor imaging) or pathological analyses (Segobin et al., 2024). Additionally, we intentionally did not correlate thalamic volumes with cortical volumes as an experimental choice, as the study was designed to characterize thalamic nuclei alterations in isolation. Forth, we only used the MMSE, BNT, AVLT, and FAB to screen cognitive functions in the present study. And we did not perform any genetic testing. However, the patients included in this study were sporadic cases, and very few patients with sporadic ALS in China carry genetic mutations, in particular patients with C9orf72 mutation (Liu et al., 2016). Finally, in this consecutive cohort, although we used age, sex, and TIV as covariates, ALS patients at King stage 3 were older than patients at King stage 1, which is consistent with some previous studies (Chiò et al., 2019; Manera et al., 2020). Similarly, in a population-based study, Manera et al. reported three regions were functionally involved in 196 patients with ALS (18.5%) at diagnosis, and 180 patients (91.8%) were older than 60 years (Manera et al., 2020). The onset of ALS appears to involve a multistep process, and aging seem to be one of the process and may accelerate the neurodegeneration of ALS. However, these findings need to be discussed by further studies.

In conclusion, our study provides a comprehensive profile of alterations in thalamic atrophy in patients with ALS. The atrophy pattern differed significantly depending on the King’s clinical disease stage, and we suggest that these alterations might largely result from sequential, regional patterns of TDP-43 pathology in ALS. Furthermore, thalamic atrophy might play pivotal roles in the motor disability and global cognitive impairments observed in patients with ALS.

## Data Availability

The raw data supporting the conclusions of this article will be made available by the authors, without undue reservation.

## Footnotes

^1^ http://surfer.nmr.mgh.harvard.edu
